# Modulation of Corneal FAK/PI3K/Akt Signaling Expression and of Metalloproteinase-2 and Metalloproteinase-9 during the Development of Herpes Simplex Keratitis

**DOI:** 10.1155/2019/4143981

**Published:** 2019-04-02

**Authors:** Lan Ke, Yanning Yang, Jing wei Li, Bo Wang, Yujing Wang, Wanju Yang, Jiangbo Yan

**Affiliations:** Department of Ophthalmology, Renmin Hospital of Wuhan University, Wuhan, Hubei, China

## Abstract

To observe the expression of MMP-2 and MMP-9 and of the FAK/PI3K/Akt signaling pathway in HSK. Fifty BALB/c mice were infected to establish the model and killed on days 0, 2, 7, 14, and 28. The cornea samples were prepared, respectively. Slit lamp examination, immunofluorescence staining, reverse transcription PCR, and Western blot were used to detect the index. After HSV-1 infection, different degrees of epithelial or stromal damage and corneal opacity were observed. Immunofluorescence staining showed that the expressions of MMP-2 and MMP-9 at different levels of corneal tissue were observed on the 0d, 2d, 7d, 14d, and 28d. Compared with 0d, the relative expression levels of MMP-2 and MMP-9 mRNA at 2d, 7d, 14d, and 28d were significantly increased (all P< 0.05). Compared with 14d, the relative expression of MMP-2 and MMP-9 mRNA decreased on the 2d, 7d, and 28d (all P< 0.05). Western blot showed that the protein expressions of p-FAK, p-PI3K, p-Akt, MMP-2, and MMP-9 at 2d, 14d, and 28d were all significantly higher than 0d (all P< 0.05). At 14 d, the expressions of p-FAK, p-PI3K, p-Akt, and MMP-2 were significantly higher than those at 2d, 7d, and 28d (all P< 0.05). The protein expression of FAK, PI3K, and Akt in corneal of mice in each time period had no significant (all P> 0.05). These data suggest that FAK/PI3K/Akt signaling pathway and MMP-2 and MMP-9 may be involved in the development of HSK.

## 1. Introduction

 Herpes stromal keratitis (HSK) is a disease that develops due to infection of the cornea with herpes simplex type 1. HSK is characterized by recurrence, and its repeated attacks can cause different degrees of damage to the corneal tissue, especially to the stroma, resulting in severe pathological damage such as corneal melting, neovascularization, secondary glaucoma, ulcer perforation, and corneal scarring [1, 2]. The incidence of HSK is approximately 1.5 million per year, of which about 40,000 people can cause severe visual impairment or corneal blindness each year [3]. However, the pathogenesis of HSK has not yet been fully clarified. Our previous study found that matrix metalloproteinase-2 and metalloproteinase-9 (MMP-2, MMP -9) play an important role in the development of HSK [4]. However, studies have not yet clearly defined which pathway leads to the secretion and expression of MMP-2 and MMP-9. Focal adhesional kinase (FAK)/phosphoinositide-3-kinase (PI3K)/protein kinase B (AKT/PKB) pathway is an important signal transduction pathway, which can regulate a variety of important biological signaling. PI3K/Akt signaling pathway has biological effects in ocular diseases, such as the occurrence and development of postcataract, choroidal neovascularization, and retinopathy of prematurity [5–7]. Recently, our group has found that FAK/PI3K/Akt signaling pathway plays an important role in keratocytes infected with HSV-1. Therefore, in this study, the HSV-1 KOS strain was used to induce the mice model of HSK. The expression of related factors, mRNA, and protein in the cornea of HSK was detected by immunofluorescence staining, RT-PCR, and Western blot. We aim to study whether the FAK/PI3K/Akt signaling pathway and MMP-2 and MMP-9 are involved in the development of HSK in the cornea to facilitate the study of the mechanism of HSK in the future.

## 2. Materials and Methods

### 2.1. Materials

#### 2.1.1. Animals

Fifty inbred male BALB/c mice of SPF grade 6~8 weeks of age and weighing 20~25 g were provided by the Experimental Animal Research Center of Hubei Province. The license number was SCXK (E) 2015-0018. Mice were bred in a temperature-controlled room at about 22~25°C with 12 h day/night cycles. The entire study complied with the ARVO statement on the use of animals in research and was approved by the Ethics Committee of the Renmin Hospital of Wuhan University.

#### 2.1.2. Main Reagents and Instruments

These included rabbit anti-mouse P-FAK antibody (ab39967, abcam); rabbit anti-mouse FAK antibody (ab61113, abcam); rabbit anti-mouse P-PI3K antibody (#4228, CST); rabbit anti-mouse PI3K antibody (#4292, CST); rabbit anti-mouse P-AKT Antibody (#4060,CST); rabbit anti-mouse AKT antibody (#4691,CST); rabbit anti-mouse MMP-2 Antibody (ab92536,abcam); mouse anti-mouse MMP-9 Antibody (ab58803, Abcam); FITC labeled goat anti-rabbit IgG (AS-1110, Aspen); CY3 labeled goat anti-mouse IgG (AS-1111, Aspen); trizol reagent (Invitrogen, USA); PrimeScript™RT Kit with gDNA Eraser, SYBR® Premix Ex Taq™ kit (TaKaRa); slicer (Shanghai Leica Instruments Co., Ltd.); ordinary optical microscope, Inverted microscope, imaging system (OLYMPUS); StepOne™ Real-Time PCR (Life technologies).

### 2.2. Methods

#### 2.2.1. Establishment of HSK Animal Model at 0d

The HSV-1 KOS strain was kindly provided by the Wuhan Virus Research Institute. The virus HSV-1 KOS strain had a titer of 2 x 10^7^ pfu/ml before use. The virus-producing cells were Vero cells (African green monkey kidney fibroblasts). Vero cells were obtained from American Type Culture Collection (ATCC). Mice were anesthetized by intraperitoneally injecting 5% chloral hydrate at a dose of 6 ml/kg. Under the microscope, we scratch the mouse corneal epithelium with the “#” mark on the back of the blade of the No. 5 surgical blade (the scratching depth needs to break through the cornea elastic layer). Subsequently, 5 *μ*l of a solution containing HSV-1 (KOS strain; 10^5^ spot forming units (pfu)) was spotted and retained for 10s on the cornea, and the eyelids were closed and massaged for 30s to allow the virus fluid to sufficiently contact the cornea. After surgery, 0.5% gentamicin eye drops were used to avoid bacterial infection. All experiments were conducted at the Center for Animal Experiment of Wuhan University.

#### 2.2.2. Slit Lamp Examination

The severity of epithelial or stromal damage and corneal opacity was assessed under the microscope at 0d, 2d, 7d, 14d, and 28d after infection with HSV-1. The evaluation index was based on the method of Heiligenhaus A[8] (Table 1).

#### 2.2.3. Immunofluorescence Staining

According to the random number table method, mice were sacrificed by cervical dislocation 0d, 2d, 7d, 14d, and 28d after infection with HSV-1 (n=2). The tissue was fixed with 4% paraformaldehyde, embedded in paraffin, and sectioned at 10 *μ*m thickness. Paraffin sections were subjected to baking, dewaxing, and antigen retrieval. After washing with PBS for 3 times, blocking with 5% BSA, removal of BSA solution, diluted MMP-2 primary antibody (1:150) and MMP-9 primary antibody (1:200) working solution were added and incubated overnight at 4°C. After washing 3 times with PBS, dilute MMP-2 secondary antibody (1:50) and MMP-9 secondary antibody (1:50) working solution were, respectively, added and incubated at 37°C for 50 min. After washing with PBS 3 times, appropriate amount of DAPI dye solution was added. Incubation was done in the dark at room temperature for 5 minutes. Finally, after washing 3 times in PBS, an appropriate amount of antifluorescence quencher was placed on the slide and the expression of MMP-2 and MMP-9 was observed under the fluorescence microscope.

#### 2.2.4. RT-PCR

According to the random number table method, mice were sacrificed by cervical dislocation 0d, 2d, 7d, 14d, and 28d after infection with HSV-1 (n=2). A small piece of tissue was placed in a solution containing 1 ml TRIzol Reagent and ground in a homogenizer. Subsequently, procedures for extracting RNA, measuring RNA concentration, reverse transcription of cDNA, and PCR amplification, are routinely performed. cDNA synthesis was performed using the PrimeScript™ RT reagent kit with gDNA Eraser (TaKaRa), and PCR amplification was performed on the StepOne™ Real-Time Cycler (Life technologies) using 3 replicate wells per sample using SYBR® Premix Ex Taq™ kit (TaKaRa) performed. The molecular primer sequence was designed as MMP-2 upstream primer: 5′- TCAACGGTCGGGAATACAGC-3′, downstream primer: 5′- AGCTGTTGTAGGAGGTGCCCT-3′, amplified fragment length: 136; MMP-9 upstream primer: 5′- AAGGGTACAGCCTGTTCCTGGT-3′, downstream primer: 5′- CTGGATGCCGTCTATGTCGTCT-3′, and amplified fragment length: 149. The experiment was repeated three times. The obtained CT value was converted by 2^-ΔΔCT^ and represents the relative expression level of mRNA.

#### 2.2.5. Western Blot

According to the random number table method, mice were sacrificed by cervical dislocation 0d, 2d, 7d, 14d, and 28d after infection with HSV-1 (n=6). The mouse corneal tissue blocks were placed in 0.4 ml tissue lysis buffer, lysed on ice, and subjected to rapid electrical homogenization. Repeat as many times as possible to crumple the tissue. The centrifuge was adjusted to 12,000 rpm at 4°C and centrifuged for approximately 10 minutes. After the supernatant was sampled for protein concentration, the protein concentration of the sample was adjusted. After centrifugation, 12% gel electrophoresis was performed. After electrotransfer of PVDF membranes, there was blocking with 5% skim milk blocking solution (TBST), anti-FAK primary antibody (1:500), p-FAK primary antibody (1:500), PI3K primary antibody (1:4000), p-PI3K primary antibody (1:1000), Akt primary antibody (1:2000), p-Akt primary antibody (1:1000), MMP-2 primary antibody (1:1000), MMP-9 primary antibody (1:1000) ). Incubate overnight at 4°C, then wash, add the corresponding secondary antibody at 4°C overnight, and develop with color. The experiment was repeated three times. The results obtained were density scanned and gray-scale analysis was performed using Gel-proAnalyzer 4.5 image analysis software.

### 2.3. Statistical Method

SPSS 22.0 statistical software was used to analyze the experimental data. All indicators are expressed as mean ± standard deviation (X±S). The t-test was used to compare the differences in the scores of different corneal indexes at different times. One-way analysis of variance was used to compare the differences in values obtained at different time points. Multiple comparisons were performed using Dunnett's test or LSD-t test. P< 0.05 was considered statistically significant.

## 3. Results

### 3.1. Clinical Course of HSK and Evaluation of Different Corneal Indexes

**Figure 1:**
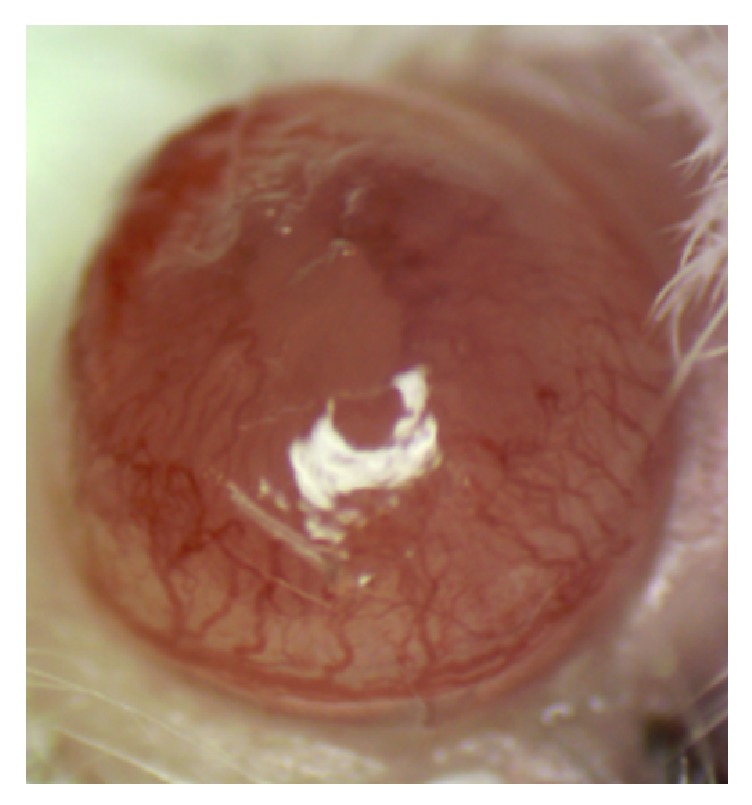
Cornea image of typical BALB/c mouse infected with HSV-1.

We found that, after infected with HSV-1, different degrees of epithelial or stromal damage, corneal opacity, and/or corneal neovascularization were observed on the 2d, 7d, 14d, and 28d. First, inflammation in corneal epithelial appeared in 1 to 3 days after corneal infection in normal BALB/c mice. The corneal epithelium showed a punctate defect or a map-like defect or a dendritic defect in the cornea. Secondly, stroma gradually developed edema after the corneal epithelium healed after 4-7 days. A few severe cases were even accompanied by corneal neovascularization, corneal ulcers, and/or corneal neovascularization. Corneal ulcers and corneal neovascularization can be seen around 14 days, and corneal ulcer perforation can occur in severe cases. Then about 28 days, the inflammation of the cornea was reduced, and corneal neovascularization became coarse and dark (Figure 1). In Table 2, we can find that, with the time of corneal infection prolonged, the score of corneal opacity gradually increased. Compared with 0d, the score of epithelial or stromal damage and corneal opacity increased in 2d, 7d, 14d, and 28d after infection, and the differences were statistically significant (P< 0.05).

### 3.2. Expression and Distribution of MMP-2 and MMP-9 Protein in Corneal Tissue at Different Time Points in HSK Mouse Model

**Figure 2:**
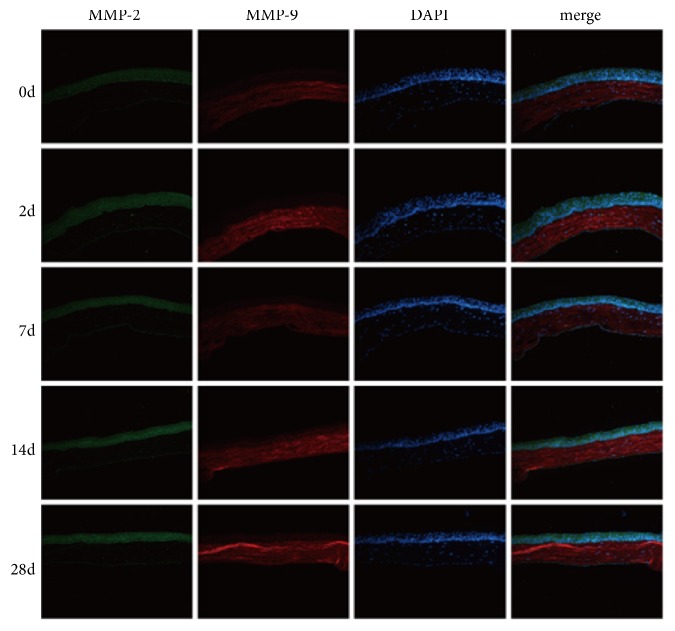
Expression of MMP-2 and MMP-9 protein in corneal tissue of BALB/c mice after 0, 2, 7, 14, and 28d of infection with HSV-1. The expression of MMP-2 in the epithelium and the expression of MMP-9 in the stroma showed diffuse fluorescence after 0d of infection. The fluorescence intensity of MMP-2 and MMP-9 at 2d was higher than that of 0d and 7d. The fluorescence intensity of MMP-2 and MMP-9 reached summit at 14d. After 28d of infection, expressions of MMP-2 and MMP-9 protein were weaker than that of 14d.

Immunofluorescence staining showed that the expression of MMP-2 and MMP-9 was observed in the corneal tissue of HSK at 0d, 2d, 7d, 14d, and 28d (Figure 2). In the corneal tissue, the expression of MMP-2 was located in the epithelium, and the expression of MMP-9 was located in the stroma of the cornea. After 2 days, the expression of MMP-2 in the corneal tissue was located in the corneal epithelium and diffused in the corneal stromal layer, while the expression of MMP-9 was located in the corneal stroma. Compared with 0d, the expression of MMP-2 and MMP-9 was stronger in 2d. After 7 days of infection with HSV-1, the expression of MMP-2 and MMP-9 in corneal tissue was stronger than that of 0d but lower than that of 2d. After 14 days, the expression of MMP-2 and MMP-9 in the corneal tissue was stronger than that of 0d, 2d, and 7d, and the intensity of fluorescence expression peaked. At 28d, the expression of MMP-2 and MMP-9 in the corneal tissue was lower than that of 14d, but it was stronger than that of 0d.

### 3.3. Expression Changes of MMP-2 and MMP-9 mRNA in Corneal Tissue at Different Time Points in HSK Mouse Model

**Figure 3:**
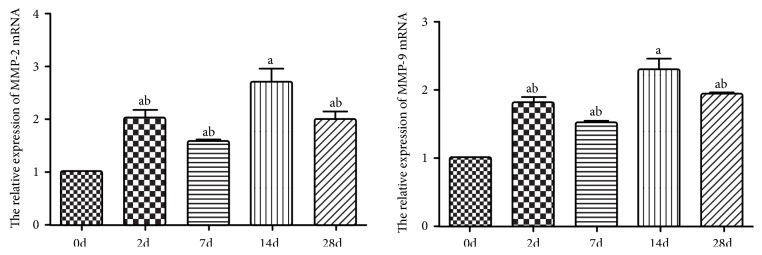
The relative expression of MMP-2 and MMP-9 mRNA in cornea at each time point. Note: ^a^P < 0.05 compared with 0d; ^b^P < 0.05 compared with 14d (One-way ANOVA, Dunnett's t test). MMP: Matrix Metalloproteinases.

The results of RT-PCR showed that the mRNA levels of MMP-2 and MMP-9 increased at 2 days after HSV infection, decreased after 7 days, then peaked again at 14 days, and then decreased at 28 days (Figure 3). The relative expression levels of MMP-2 mRNA at 0 d, 2 d, 7 d, 14 d, and 28 d after infection in HSK mouse model were 1.00±0.00, 1.59±0.04, 2.02±0.15, 2.70±0.26, and 1.99±1.86, respectively. The relative expression levels of MMP-9 mRNA were 1.00±0.00, 1.51±0.05, 1.81±0.07, 2.30±0.15, and 1.94±1.71. Compared with 0d, the relative expression levels of MMP-2 and MMP-9 mRNA were significantly increased at 2d, 7d, 14d, and 28d, and the differences were statistically significant (all P< 0.05). Compared with 14d, the relative expression levels of MMP-2 and MMP-9 mRNA decreased at 2d, 7d, and 28d, and the differences were statistically significant (all P< 0.05) (Table 3).

### 3.4. Expression of p-FAK, FAK, p-PI3K, PI3K, p-Akt, Akt, MMP-2, and MMP-9 in Corneal Tissue at Different Time Points in HSK Mouse Model

**Figure 4:**
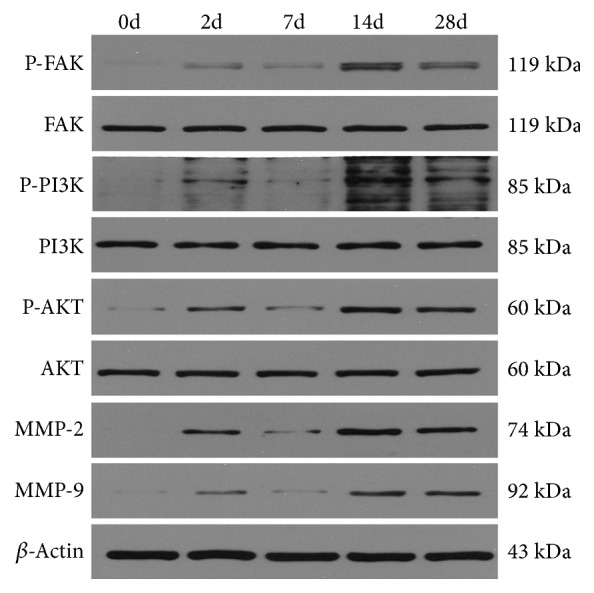
Western Blot results of p-FAK, FAK, p-PI3K, PI3K, p-Akt, Akt, MMP-2, and MMP-9 protein expression in mouse cornea at each time point.

**Figure 5:**
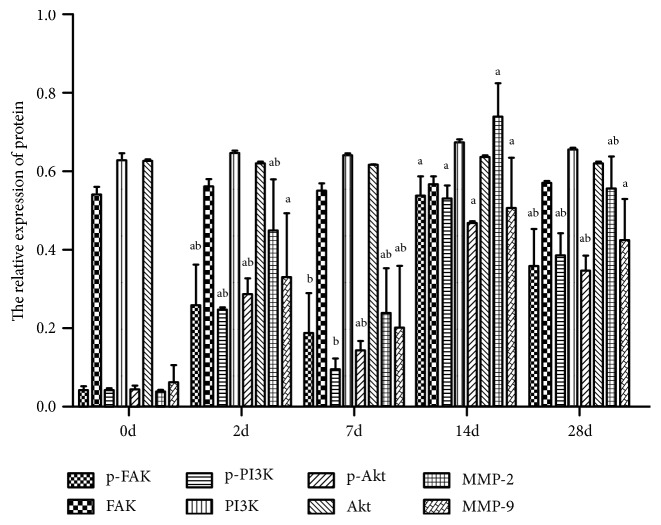
Relative expression of p-FAK, FAK, p-PI3K, PI3K, p-Akt, Akt, MMP-2, and MMP-9 in cornea at each time point.* Note. *^a^P < 0.05 compared with 0d; ^b^P < 0.05 compared with 14d (one-way ANOVA, LSD-t test).

Western blot analysis showed that the expression of p-FAK, p-PI3K, p-Akt, MMP-2, and MMP-9 was increased in corneal tissue at 2 days after infection and decreased from 7d to 2d, 14d, and 28d. It rose again and peaked at 14d (Figures 4 and 5). The protein expressions of p-FAK, p-PI3K, p-Akt, MMP-2, and MMP-9 at 2d, 14d, and 28d were all significantly higher than those at 0d (P< 0.05). The expression of p-Akt, MMP-2, and MMP-9 at 7 days after infection was significantly higher than that at 0 days (P< 0.05). At 14 days, the expression of p-FAK, p-PI3K, p-Akt, and MMP-2 protein was higher than that at 2d, 7d, and 28d, and the difference was statistically significant (P< 0.05). The expression of MMP-9 protein at 7d was lower than that at 14d, and the difference was statistically significant (P< 0.05). There was no significant difference in the expression levels of FAK, PI3K, and Akt between tissues at all time points (P> 0.05) (Table 4).

## 4. Discussion

HSK is one of the leading eye diseases in the world leading to corneal blindness [3]. Recurrent HSK will undoubtedly aggravate the deterioration of the disease and eventually lead to blinding outcomes. Although the research on molecular mechanisms related to the formation of HSK is increasing and deepening, the pathogenesis of HSK is complex and its pathogenesis is still not fully understood. There is no clinically satisfactory drug treatment that can prevent it from recurring or prevent it from developing into a bad outcome. Therefore, it is imperative to explore drug treatment with multiple pathways, multiple targets, and multiple therapeutic effects.

Recent research indicates that FAK not only participates in the regulation of cell proliferation, apoptosis, migration, invasion, metastasis, and adhesion but also participates in the regulation of the expression and activity of MMPs and TIMPs through multiple signaling pathways [9–12]. At the same time, our previous study found that there were two peaks in the phosphorylation of FAK in the corneal epithelium of HSV-1-infected mouse corneal models. The phosphorylation levels of FAK increased on the 2d, 14d, and 28d after infection and decreased on the 7d compared with 2d [13]. The expression pattern of MMP-2 and MMP-9 in the corneal model of HSV-1-infected mice in this study is very similar to our previous studies [4]. In addition, we also found that TNF-*α* can increase the expression and secretion of MMP-2 and MMP-9 through the FAK/ERK signaling pathway in vitro experiments [14]. This further suggests the role of the FAK signaling pathway in HSK.

PI3K/Akt is an important signaling pathway downstream of FAK that regulates many important biological signaling pathways. It has been reported that, on the one hand, the herpes virus can enhance its function of replication, transcription, and translation by regulating the PI3K/Akt signaling pathway. On the other hand, the PI3K/Akt signaling pathway can be used to facilitate virus entry, replication, delay, and activation [15–17]. Activation of PI3K/Akt signaling can occur at multiple stages of the herpes virus life cycle [16, 17]. Cheshenko reported that HSV can activate and release intracellular calcium and phosphorylate FAK via the integrin signaling pathway, which activates the AKT signaling pathway and provides suitable conditions for virus entry into cells [18]. Therefore, we hypothesized that the FAK/PI3K/Akt signaling pathway and MMP-2 and MMP-9 may be involved in the development of HSK in the cornea.

In this study, we first studied the patterns of FAK, p-FAK, PI3K, p-PI3K, Akt, and p-Akt in the FAK/PI3K/Akt signaling pathway and MMP-2 and MMP-9 in the HSK mouse model. By Western blot, we observed that the levels of p-FAK, p-PI3K, and p-Akt were upregulated on the 2d, 7d, 14d, and 28d after infection. The upregulated level on the 7th day decreased compared with that on the 2d day and reached the maximum on the 14d day. At the same time, the changes in the levels of MMP-2 and MMP-9 protein were similar to those of p-FAK, p-PI3K, and p-Akt. This is similar to our previous study [4], suggesting a potential link between the FAK/PI3K/Akt signaling pathway and the MMP-2 and MMP-9. In addition, we have further demonstrated by RT-PCR and Immunofluorescence staining that the expression and activation of MMP-2 and MMP-9 in the mouse cornea at the early stage of HSV-1 infection play an active role in the development of HSK. To further elucidate the relationship between the FAK/PI3K/Akt signaling pathway and the HSK, we plan to use the inhibitor.

In summary, our results provide in vivo evidence that FAK/PI3K/Akt signaling pathway and MMP-2 and MMP-9 may be involved in the development of HSK. The discovery of the association between FAK/PI3K/Akt signaling pathway, MMP-2 and MMP-9, and HSK may facilitate the study of the mechanism of HSK, and studies of the FAK/PI3K/AKT signaling pathway may provide new targets for the treatment of HSK. However, in order to show the regulatory relationship between FAK/PI3K/Akt signaling pathway and MMP-2 and MMP-9, further research is necessary.

## Figures and Tables

**Table 1:** Evaluate different indexes of cornea in HSV-1 infected mice.

Score	Epithelial or stromal damage	Corneal opacity
0	-	Transparent

1+	<25%	Slightly turbid

2+	≥25%, <50%	Moderately turbid, visible iris

3+	≥50%, <75%	Severe turbidity, pupillary margin can be judged

4+	≥75%	Complete opacity, invisible posterior features

**Table 2:** Evaluate different indexes of cornea in HSV-1 infected mice (*X*±*s).*

Indexes	0d	2d	7d	14d	28d
Epithelial or stromal damage	0.00±0.00	1.03±0.16^a^	1.00±0.37^a^	1.50±0.69^a^	1.60±0.84^a^

Corneal opacity	0.00±0.00	1.03±0.16^a^	1.03±0.32^a^	2.20±0.41^a^	2.50±0.71^a^

*Note.* Compared with 0d, ^a^P < 0.05 (t test).

**Table 3:** The relative expression of MMP-2 and MMP-9 mRNA in the cornea at each time point (*X*±*s*).

Time point	MMP-2	MMP-9
0d	1.00±0.00	1.00±0.00
2d	2.02±0.15^ab^	1.81±0.07^ab^
7d	1.59±0.04^ab^	1.51±0.05^ab^
14d	2.70±0.26^a^	2.30±0.15^a^
28d	1.99±0.15^ab^	1.94±0.02^ab^

F	50.69	122.16
P	<0.05	<0.05

*Note.* ^a^P < 0.05 compared with 0d; ^b^P < 0.05 compared with 14d (One-way ANOVA, Dunnett's t test).

MMP: matrix metalloproteinases.

**Table 4:** Comparison of relative expression levels of p-FAK, FAK, p-PI3K, PI3K, p-Akt, Akt, MMP-2, and MMP-9 in cornea at each time point (*X*±*s).*

Time point	p-FAK	FAK	p-PI3K	PI3K	p-Akt	Akt	MMP-2	MMP-9
0d	0.04±0.01	0.54±0.02	0.04±0.01	0.63±0.02	0.04±0.01	0.63±0.00	0.04±0.00	0.06±0.04
2d	0.26±0.10^ab^	0.56±0.02	0.24±0.01^ab^	0.64±0.01	0.29±0.04^ab^	0.62±0.01	0.45±0.13^ab^	0.33±0.16^a^
7d	0.19±0.10^b^	0.55±0.02	0.09±0.03^b^	0.64±0.01	0.14±0.02^ab^	0.61±0.00	0.24±0.11^ab^	0.20±0.15^ab^
14d	0.54±0.05^a^	0.57±0.02	0.53±0.03^a^	0.67±0.00	0.47±0.01^a^	0.64±0.01	0.74±0.09^a^	0.50±0.13^a^
28d	0.35±0.10^ab^	0.57±0.01	0.39±0.05^ab^	0.66±0.01	0.35±0.04^ab^	0.63±0.00	0.56±0.08^ab^	0.42±0.10^a^

F	15.51	1.51	123.38	8.73	119.07	10.14	25.31	5.84
P	<0.05	>0.05	<0.05	>0.05	<0.05	>0.05	<0.05	<0.05

*Note.* ^a^P < 0.05 compared with 0d; ^b^P < 0.05 compared with 14d (one-way ANOVA, LSD-t test).

## Data Availability

The data used to support the findings of this study are currently kept under raps while the research findings are significant. Requests for data, 12 months after publication of this article, will be considered by the corresponding author on reasonable request.
